# Insecticide Resistance Status of United States Populations of *Aedes albopictus* and Mechanisms Involved

**DOI:** 10.1371/journal.pone.0101992

**Published:** 2014-07-11

**Authors:** Sébastien Marcombe, Ary Farajollahi, Sean P. Healy, Gary G. Clark, Dina M. Fonseca

**Affiliations:** 1 Center for Vector Biology, Rutgers University, New Brunswick, New Jersey, United States of America; 2 Mercer County Mosquito Control, West Trenton, New Jersey, United States of America; 3 Monmouth County Mosquito Extermination Commission, Eatontown, New Jersey, United States of America; 4 Mosquito and Fly Research Unit, Agriculture Research Service, United States Department of Agriculture, Gainesville, Florida, United States of America; Virginia Tech, United States of America

## Abstract

*Aedes albopictus* (Skuse) is an invasive mosquito that has become an important vector of chikungunya and dengue viruses. Immature *Ae. albopictus* thrive in backyard household containers that require treatment with larvicides and when adult populations reach pest levels or disease transmission is ongoing, adulticiding is often required. To assess the feasibility of control of USA populations, we tested the susceptibility of *Ae. albopictus* to chemicals representing the main insecticide classes with different modes of action: organochlorines, organophosphates, carbamates, pyrethroids, insect growth regulators (IGR), naturalytes, and biolarvicides. We characterized a susceptible reference strain of *Ae. albopictus*, ATM95, and tested the susceptibility of eight USA populations to five adulticides and six larvicides. We found that USA populations are broadly susceptible to currently available larvicides and adulticides. Unexpectedly, however, we found significant resistance to dichlorodiphenyltrichloroethane (DDT) in two Florida populations and in a New Jersey population. We also found resistance to malathion, an organophosphate, in Florida and New Jersey and reduced susceptibility to the IGRs pyriproxyfen and methoprene. All populations tested were fully susceptible to pyrethroids. Biochemical assays revealed a significant up-regulation of GSTs in DDT-resistant populations in both larval and adult stages. Also, β-esterases were up-regulated in the populations with suspected resistance to malathion. Of note, we identified a previously unknown amino acid polymorphism (Phe → Leu) in domain III of the VGSC, in a location known to be associated with pyrethroid resistance in another container-inhabiting mosquito, *Aedes aegypti* L. The observed DDT resistance in populations from Florida may indicate multiple introductions of this species into the USA, possibly from tropical populations. In addition, the mechanisms underlying DDT resistance often result in pyrethroid resistance, which would undermine a remaining tool for the control of *Ae. albopictus*. Continued monitoring of the insecticide resistance status of this species is imperative.

## Introduction

*Aedes* (*Stegomyia*) *albopictus* (Skuse), the Asian tiger mosquito, is an aggressive human- and day-biting species native to Asia that has recently expanded to at least 28 countries outside its native range, and now occurs in all inhabitable continents [1]. Detailed theoretical analyses indicate that the spread of *Ae. albopictus* may well continue into many more regions of the world [1]–[3]. Although this species is often considered mostly an urban nuisance, it was the principal dengue vector in Hawaii and other areas were *Aedes aegypti* L. populations have been controlled [4] and in the summer of 2013, an autochthonous case of dengue in Suffolk County, New York has been attributed to thriving populations of *Ae. albopictus* [5]. Furthermore, since recent mutations in the chikungunya virus (CHIKV) increased the vector competence of *Ae. albopictus* for the viral agent [6], [7], chikungunya has become epidemic in Africa and the Indian Ocean Basin [8]. Although chikungunya fever has not spread broadly in the temperate zone, an epidemic in northern Italy in 2007 sickened over 200 people [9] and small numbers of locally transmitted CHIKV cases were identified in southern France in 2010 [10], both of which were driven by local populations of *Ae. albopictus*. The European expansion of CHIKV would not have been possible without the prior invasion of that continent by *Ae. albopictus* [11].

*Aedes albopictus* is a container-inhabiting mosquito strongly associated with human habitats (especially outside its native range) and capable of ovipositing diapause-destined eggs that survive even in cold northern latitudes in parts of its native (*e.g*., northern Japan, China) and introduced (*e.g*., Europe and northeastern USA) ranges [12]. The first line of control against *Ae. albopictus* is often source reduction [13], but when containers cannot be removed or emptied, larvicides are used [13]. If adults become a serious nuisance, or disease outbreaks are ongoing or imminent, insecticides targeting the adults are applied [14].

Unfortunately, the development and spread of insecticide resistance represents a serious threat as it can lead to a reduction of the efficacy of larvicide or adulticide-based control programs, as demonstrated in the control of the main dengue vector *Ae. aegypti* [15], [16]. In contrast to *Ae. aegypti*, there have been only a few reports of insecticide resistance in *Ae. albopictus* worldwide [16], [17]. Several studies implemented in the 1960s and summarized by Mouchet et al. [18] showed that several populations of *Ae. albopictus* from Southeast Asia and India were resistant to some of the insecticides used at the time for vector control (*i.e*., DDT, dieldrin and fenthion). A recent review by Ranson et al. [16] updated by Vontas et al. [19] summarized the levels of insecticide resistance in *Ae. albopictus* worldwide. It is apparent that resistance to the main families of insecticides currently or historically used for vector control across the world (*i.e*., DDT, organophosphates and pyrethroids) has been found in *Ae. albopictus* [20]–[25]. In the USA, to our knowledge, only four studies have reported on insecticide resistance in *Ae. albopictus*: one population in Florida was resistant to the organophosphate malathion [26], populations in Texas and Illinois were also resistant to malathion [25], [27], and resistance to a pyrethroid (deltamethrin) was found in a population from Alabama [28].

Insecticide resistance can be associated with mutations in the sequence of the target protein that induce insensitivity to the insecticide (target-site resistance), and/or to the up-regulation of detoxification enzymes (metabolic-based resistance). The main target site resistance mechanisms known in mosquitoes involve 1) amino acid substitutions in the voltage gated sodium channel that cause a resistance phenotype to pyrethroid (DDT) insecticides known as knockdown resistance (Kdr, [29] and 2) mutations in the acetylcholine esterase sequence that lead to insensitivity of this enzyme to organophosphates [30]. Metabolic-based resistance involves the bio-transformation of the insecticide molecule by enzymes and is now considered a key resistance mechanism in insects [31], [32]. Three large enzyme families, the cytochrome P450 monooxygenases (P450s), glutathione S-transferases (GSTs), and carboxy/cholinesterases (CCEs) have been implicated in the metabolism of insecticides [32]–[34]. So far, compared to other mosquito species of importance such as *Anopheles* spp., *Culex* spp., and *Ae. aegypti*, very little is known about the molecular or biochemical basis of resistance in *Ae. albopictus* and, in particular, to our knowledge, no studies have specifically examined the underlying mechanisms of resistance in USA *Ae. albopictus*.

The objective of the present study was to determine the insecticide resistance status of *Ae. albopictus* across the full latitudinal range of the species in the USA. Specifically, we examined populations from New Jersey, Pennsylvania, and Florida (Table 1). We chose eleven chemicals that represent the main classes of insecticides historically or currently used for mosquito control (Table 2), including some that have only recently been adopted. We compared the levels of resistance of field-collected specimens to a susceptible strain of *Ae. albopictus* that we characterized for this purpose (reference strain ATM95). In addition, we used biochemical and molecular assays to identify putative resistance mechanisms in *Ae. albopictus* such as target-site mutations and up-regulation of detoxifying enzymes.

**Table 1:** Detailed description with geographic and socio-economic information of the sources of mosquito populations.

State	County	Municipality	Mosquito population name abbreviations	Coordinates	Altitude	Human density inhabitants/Km^2^
New Jersey	Bergen	Elmwood Park	NJBer	40°54′N74°70′W	14 m	2,829
	Mercer	Trenton	NJMer1	40°13′N74°45′W	15 m	4,286
		Ewing	NJMer2	40°15′N74°47′W	38 m	906
	Monmouth	Middletown	NJMon1	40°24′N74°04′W	30 m	626
		Belmar	NJMon2	40°10′N74°01′W	4 m	2,140
Pennsylvania	York	York	PA	39°57′N76°43′W	121 m	3,061
Florida	St John's	St Augustine south	FL1	29°50′N81°18′W	7 m	1,118
		St Augustine Beach	FL2	29°53′N81°18′W	0 m	936

Population name abbreviations are used throughout the text.

**Table 2:** Name, class, and mode of action of all insecticides tested in this study.

Status	Insecticide	Family	Mode of action
**Larvicide**	*Bti*	Biolarvicide	Cell membrane destruction
	Spinosad	Naturalyte	Nicotinic acetylcholine receptor
	Temephos	Organophosphate	Acetylcholinesterase inhibitor
	Propoxur	Carbamate	
	Methoprene	Insect Growth Regulator	Juvenile hormone mimics
	Pyriproxyfen		
**Adulticide**	Malathion	Organophosphate	Acetylcholinesterase inhibitor
	DDT	Organochlorine	Sodium channel modulator
	Deltamethrin	Pyrethroid	
	Prallethrin		
	Phenothrin		

## Materials and Methods

### Ethics statement

No specific permits were required for collection of field specimens, which were performed in urban and suburban backyards in the US states of New Jersey, Pennsylvania, and Florida with homeowners assent by professional county mosquito control personnel. These studies did not involve endangered or protected species. In the laboratory, mosquito colonies were blood fed on quail, *Colinus virginianus*, under the guidelines of the Rutgers University Animal Use Protocol# 86–129 that was approved by the Rutgers IACUC.

### Mosquito strains and collection

**Figure 1:**
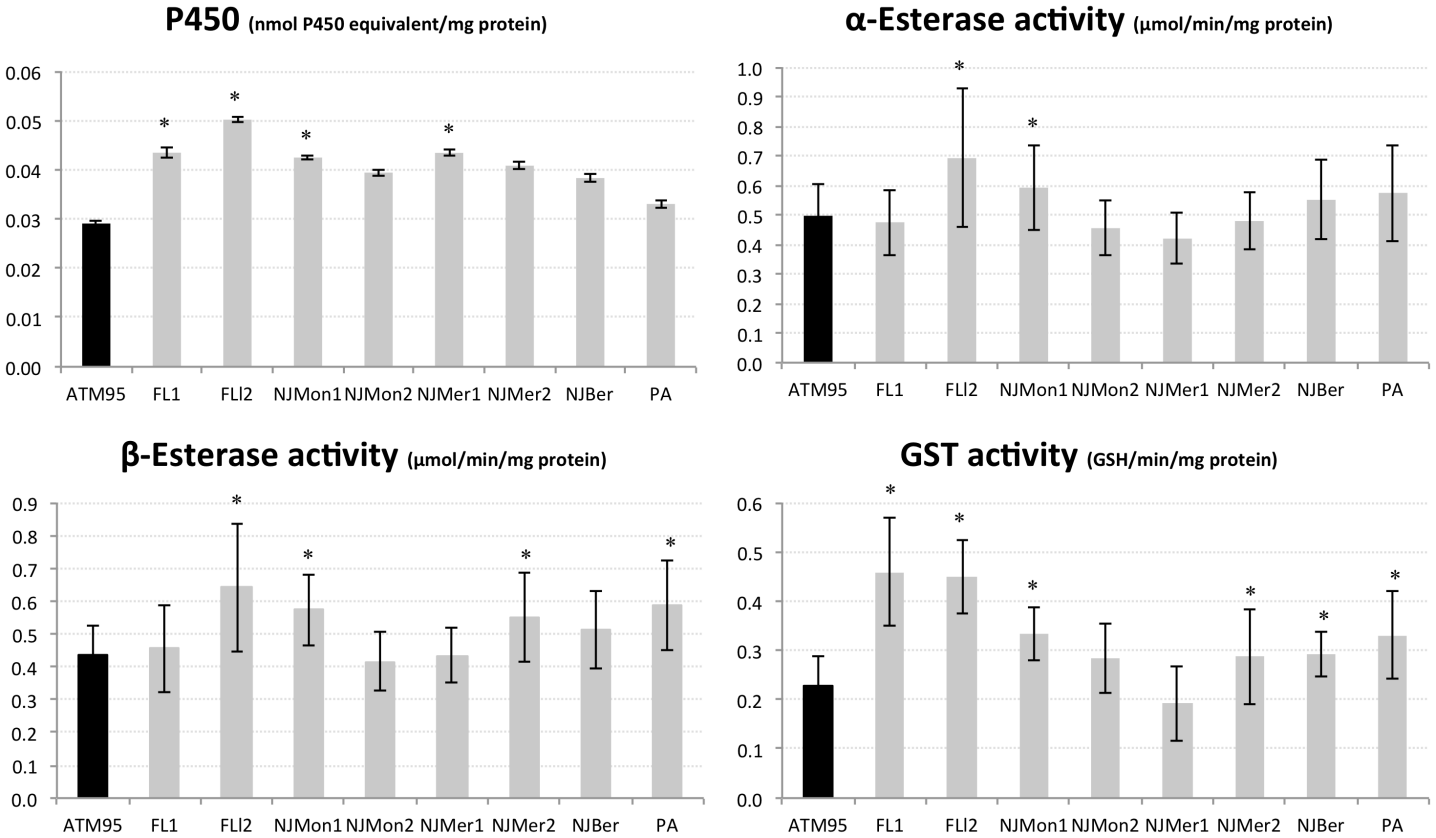
Global amount or activity of detoxification enzymes in *Aedes albopictus* larvae from field populations and the laboratory strain (ATM95): cytochrome P450 monooxygenases (P450s), Esterase (α and β-CCEs), and Glutathione-S transferases (GSTs). Sample sizes are 47 specimens/population (15 for P450, n = 3). Confidence intervals are one standard deviation of the mean. An asterisk (*) denotes significantly up-regulated values compared to the susceptible reference strain ATM95, Tukey-Kramer test.

We characterized a reference laboratory strain (ATM95) and tested eight field populations of *Ae. albopictus* (Table 1). *Aedes albopictus* was first detected in New Jersey (NJ) on August 1, 1995 in a standard NJ light trap collection in Keyport [35]. Surveillance at a marina 300 m from the trap site yielded *Ae. albopictus* larvae from one discarded bucket and 2 tires and a colony started from this population, now named ATM95, has been continuously reared in the laboratory at the Center for Vector Biology at Rutgers University in New Brunswick, NJ without exposure to insecticides. Preliminary bioassays on the ATM95 strain showed that this strain could be considered susceptible in comparison to previous results from the literature. The field caught *Ae. albopictus* samples were collected as larvae, pupae, or eggs (ovitraps) in one site in Bergen county, NJ (NJBer, N 40°47′33′′, W 74°1′32′′), two replicate sites (less than 5 km apart) in Mercer county, NJ (NJMer1, NJMer2, N 40°13′1′′ W 74°44′35′′), two sites in Monmouth county, NJ (NJMon1 and NJMon2, N 40°26′36′′ W 74°13′5′′), one site in York county, Pennsylvania (PA, N 39°57′46′′ W 76°43′41′′) and two sites in St. Johns county, Florida (FL1 and FL2, N 29°53′39′′ W 81°18′48′′) during the 2011 active mosquito season (Figure 1). All stages were reared to adults in the laboratory on a diet of powdered cat food. After emergence of female *Ae. albopictus* they were provided restrained quails (*Colinus virginianus*) as sources of blood for egg development following the Rutgers University Animal Use protocol# 86–129. Larvae and adults obtained from the F_1_ progeny were used for bioassays and biochemical and molecular studies.

### Bioassays

We chose to test the susceptibility of *Ae. albopictus* to a range of insecticides representative of those historically and currently used for mosquito control in the USA from all main families of insecticides with different modes of action (Table 2).

#### Larval bioassays

Larval bioassays were carried out using the water-dispersible granule formulation (VectoBac WDG, Valent BioSciences, Libertyville, IL, USA) of *Bacillus thuringiensis* var. *israelensis* (*Bti*) (37.4% ai, 3000 ITU/mg). The remaining insecticides were tested by diluting the active ingredients (ai) purchased from Sigma-Aldrich (Seelze, Germany) in ethanol to required levels according to WHO guidelines [36]. We tested temephos (97.3% active ingredient [ai]), propoxur (99.8%), spinosad (97.6%), methoprene (95.6%), and pyriproxyfen (99.1%). All bioassays were performed using late third and early fourth-instars of *Ae. albopictus*.

To determine the activity range of the larvicides in *Ae. albopictus*, larvae of the susceptible laboratory strain, ATM95, were exposed to 3 replicates of a wide range of test concentrations. For each bioassay, 25 larvae of each population were transferred to plastic cups containing 99 mL of distilled water with 1 mL of the insecticide at the desired concentration. The appropriate volume of dilution from the stock solution was added to the water in the cups to obtain the desired target dosage, starting with the lowest concentration. Four cups per concentration (100 larvae) and 4 to 8 concentrations in the activity range of the insecticide (between 10% and 95% mortality) were used to determine LC_50_ and LC_90_ values (LC: lethal concentration). Control treatments were made with 99 mL of distilled water and 1 mL of ethanol. Larval mortality was recorded after 24 h exposure except for pyriproxyfen and methoprene for which mortality was recorded every 24 h until emergence due to the delayed action of these insect growth regulators. In this case, larvae were provided with food at a concentration of 100 mg/L every day. For each bioassay, temperature was maintained at 27°C in an incubator with a 16L:8D photoperiod.

#### Adult bioassays

Adult bioassays also followed WHO protocols [37], with 3 to 5 day old females of each F_1_ progeny used for tarsal contact tests with insecticide-treated filter paper and compared with the susceptible ATM95 strain. We started with technical grade (Pestanal Sigma-Aldrich, Seelze, Germany) deltamethrin (99.7% ai, type II pyrethroid), prallethrin (96.2%, type I), phenothrin (94.4%, type I), malathion (97.2%), and DDT (99.7%). Insecticide was applied to filter paper by dripping evenly onto the paper 2 mL of technical grade chemical dissolved in acetone and silicone oil to the appropriate concentration [37]. Concentrations were expressed in w/w percentage of the active ingredient in silicone oil. Filter papers were dried for 24 h before the test. The resistance status of *Ae. albopictus* populations from each locality was determined by using WHO discriminating dosages (DD; double concentration of LC_99_) of deltamethrin (0.05%), malathion (0.8%), and DDT (4%). Preliminary bioassays conducted on the ATM95 strain displayed that the discriminating dosages for prallethrin and phenothrin were 1% and 1.5%, respectively. Those two pyrethroids are used in combination in the newly available Duet dual-action adulticide formulation (Clarke Mosquito Control, Roselle, Illinois, USA) for adult mosquito control. For each strain, five batches of 20 non-blood fed females (2–5 days old; n = 100) were exposed to the insecticides in WHO test kits for 60 min to estimate the knock down effect (KDT_50_ and KDT_90_) of the insecticides. The number of knocked down mosquitoes in the tubes was counted every 2 minutes. The adults were then transferred into holding tubes, were provided with sugar solution (10%), and kept at 27°C with a relative humidity of 80%. Mortality was recorded 24 h later. Mosquitoes exposed for 1 h to paper impregnated with the carrier (silicone oil) mixed with acetone were used as controls. Tests were replicated twice when the number of available mosquitoes was suitable. Following WHO criteria a population is considered resistant if the mortality after 24 h is under 90%, resistance is suspected with mortality between 90 and 98% and a population is susceptible with mortality over 98%.

Larval and adult knock down times (KDT) were analyzed with the log-probit method of Finney [38] using the Sakuma Probit software [39]. Data from all replicates were pooled for analysis. Lethal concentrations (LC_50_ and LC_95_ for larvae) and knock-down time (KDT_50_ and KDT_95_ for adults) were calculated together with their 95% confidence intervals. Adult mortality after 24 h exposure was also recorded for each population. Compared to the susceptible ATM95 strain field populations were considered as having some resistance to a given insecticide when their LC_50/95_ or KDT_50/95_ ratios (resistance ratio: RR_50/95_) had confidence limits that excluded the value 1. We considered resistance to be moderate to strong when RR_50/95_ values rose above 2.

### Biochemical assays

The levels of P450 monooxygenases (P450s), and the activities of carboxy/cholinesterases (CCEs) and glutathione S-transferases (GSTs) were assayed from single 3 days-old F_1_ females (n = 47) following microplate methods described by Hemingway [32] and Brogdon [40] on an Epoch spectrophotometer (BioTek, Vermont, USA). Total protein quantification of mosquito homogenates was performed using Bradford reagent with bovine serum albumin as the standard protein [41] to normalize enzyme activity levels by protein content. For P450 assays, the OD values were measured at 620 nm after 30 min incubation of individual mosquito homogenate with 200 µL of 2 mM 3, 3', 5, 5'-tetramethylbenzidine dihydrochloride (TMBZ) and 25 µL of 3% hydrogen peroxide and the quantity was determined from cytochrome-c standard curve. Nonspecific α- and β-CCEs activities were assayed by 10 min incubation of mosquito homogenate in each well with 100 µL of 3 mM napthyl acetate (either α- or β-) at room temperature and the OD values were measured at 540 nm. The activity was determined from α- or β-naphtol standard curves. Glutathione-S-transferases activity was measured in the reaction containing 2 mM reduced glutathione and 1 mM 1-chloro-2,4-dinitrobenzene (CDNB). The reaction rates were measured at 340 nm after 20 min, and the activity was expressed in nmoles GSH conjugated/min/mg protein.

Statistical comparisons of detoxification enzyme levels between ATM95 and the field populations were assessed with Tukey-Kramer tests in JMP8.0.1 (SAS Institute, Cary, North Carolina, USA) using a P value threshold of 0.05. Tukey-Kramer HSD (honestly significant difference) test is a highly conservative test that accounts for multiple comparisons [42].

### Kdr genotyping

We extracted DNA from 14 adult *Ae. albopictus* collected in Florida (FL1 and FL2) using DNAeasy tissue kits (Qiagen, Valencia, California, USA). We chose 6 survivors and 6 dead specimens following DDT exposure and amplified portions of domains II, III, and IV of the voltage-gated sodium channel (VGSC), a known target of DDT and pyrethroid insecticides, using primers from Kasai et al. [43]. Specifically we amplified and sequenced domain II with aegSCF20 and aegSCR21, domain II with aegSCF7 and aegSCR8, and domain IV with albSCF6 and albSCR8. Our PCR was composed of 1× PCR buffer, 2.5 mM of MgCl_2_ (2.0 mM for Domain III), 200 µM of each dNTP, 0.2 mg/ml of BSA, 0.2 µM of each primer, and 1 unit of *TaqGold* (Applied Biosystems, Foster City, California, USA). The PCR cycle started with a 10 min denaturation (and *TaqGold* activation) at 96°C followed by 40 cycles of 30 s at 96°C, 30 s at 55°C (Domain II and IV) or 53°C (Domain III) and 45 s at 72°C, and a final extension of 10 min at 72°C. The PCR products were cleaned with ExoSAP-IT (USB, Cleveland, Ohio, USA) and cycle sequenced for analyses on an ABI 3100 automated sequencer (Applied Biosystems). Sequences were cleaned and checked with Sequencher 5.0 (Gene Codes, Ann Harbor, Michigan, USA).

### Enzymatic phenotyping of Ache1

The phenotypes of the acetylcholine esterase AChE1, encoded by the ace-1 gene, were examined in each population (n = 24) using the previously described TDP test [44] adapted for *Ae. albopictus* with both dichlorvos and propoxur concentrations of 1.10^−2^ M. The TDP test identifies all possible phenotypes containing the G119S, F290V and wild-type (susceptible) alleles.

## Results

### Larval and adult bioassays

Larval bioassays resulted in low resistant ratios (RRs) indicating that none of the eight USA populations of *Ae. albopictus* were resistant to the larvicides tested (Table 3). However, one of the populations from Florida, FL2, showed significant resistance to both methoprene and pyriproxyfen (IGRs) with RRs of 3.72 and 2.36 fold, respectively. Further, all the populations had values of RRs for propoxur that excluded 1, ranging from 1.47 (NJMon1) to 2.8 fold (FL1 and FL2); the latter indicating significant resistance to this carbamate in Florida populations. The insecticidal activities of the larvicides used against the ATM95 strain (Table 3) can be ranked as follows: pyriproxyfen > methoprene > temephos > *Bti* > spinosad > propoxur with LC_50_ of 9.4E-6, 1.4E-4, 5.4E-3, 0.07, 0.1 and 1.02 mg/L, respectively.

**Table 3:** Resistance status of larvae *Aedes albopictus*.

	*Bti*		Temephos		Propoxur		Spinosad		Methoprene		Pyriproxyfen	
Population	LC_50_ (95% CI)	RR_50_	LC_50_ (95% CI)	RR_50_	LC_50_ (95% CI)	RR_50_	LC_50_ (95% CI)	RR_50_	LC_50_ (95% CI)	RR_50_	LC_50_ (95% CI)	RR_50_
**ATM95**	0.07 (0.066–0.071)	1	5.4E-03 (5.1E-03-5.7E-03)	1	1.02 (0.93–1.09)	1	0.10 (0.036–0.106)	1	1.4E-04 (9.9E-05-1.7E-04)	1	9.4E-06 (3.6E-06-2.5E-05)	1
**FL1**	0.07 (0.01–0.07)	0.99	5.3E-03 (4.9E-03-5.3E-03)	0.93	2.87 (2.67–3.15)	**2.82**	0.15 (0.145–0.162)	1.51	-	-	1.5E-05 (1.2E-05-1.9E-05)	1.57
**FL2**	0.06 (0.043–0.085)	0.84	5E-03 (5E-03-5.6E-03)	0.99	2.83 (2.59–3.29)	**2.77**	0.16 (0.151–0.165)	1.56	5.1E-04 (3.1E-04-1E-03)	**3.72**	2.2E-05 (1.7E-05-2.9E-05	**2.36**
**NJMon1**	0.12 (0.108–0.145)	**1.78**	4.7E-03 (4.5E-03-4.9E-03)	0.87	1.50 (1.32–1.79)	**1.47**	0.14 (0.138–0.147)	1.42	1.6E-04 (1.2E-04-2.1E-04)	1.15	4.7E-06 (2.6E-06-6.5E-06)	**0.50**
**NJMon2**	0.11 (0.10–0.13)	**1.68**	6.1E-03 (5.7E-03-6.6E-03)	**1.14**	1.62 (1.44–1.9)	**1.59**	0.10 (0.091–.112)	1.01	7.4E-05 (3.1E-05-1.2E-4)	**0.54**	3.6E-06 (2.2E-06-8.3E-06)	**0.38**
**NJMer1**	0.08 (0.06–0.1)	1.16	6.1E-03 (5.6E-03-7E-03)	**1.13**	1.73 (1.62–1.89)	**1.69**	0.14 (0.109–0.29)	1.38	1.7E-04 (1E-4-2.6E-04)	**1.22**	5.7E-06 (3.3E-06-8.2E-06)	**0.60**
**NJMer2**	0.08 (0.073–0.089)	**1.19**	6.3E-03 (5.8E-03-6.8E-03)	**1.17**	2.09 (1.68–3.26)	**2.05**	0.08 (0.068–0.089)	0.79	9.9E-05 (8.8E-6-3.E-04)	0.71	1.3E-05 (9.5E-06-1.7E-05)	1.37
**NJBer**	0.05 (0.047–0.057)	**0.76**	6.9E-03 (6.4E-03-7.4E-03)	**1.27**	2.13 (1.83–2.82)	**2.09**	0.16 (0.142–0.189)	1.56	4.5E-05 (1.4E-05-8.4E-05)	**0.33**	1.7E-05 (1.2E-05-2.4E-05)	**1.81**
**PA**	0.08 (0.069–0.085)	**1.13**	7.6E-03 (6.8E-03-8.8E-03)	**1.41**	1.94 (1.66–2.41)	**1.90**	0.18 (0.144–0.309)	1.73	1.1E-04 (5.7E-05-2.3E-04)	0.78	1.0E-05 (7.8E-06-1.4E-05)	1.11

ATM95: susceptible reference strain; LC_50_: Lethal Concentration that kills 50% of the population (mg/L); RR_50_: Resistant Ratio  =  LC_50_ susceptible strain (ATM95)/LC_50_ field population; CI: Confidence Interval. Significant RRs are shown in bold (P<0.05).

The knockdown times (KDT) for *Ae. albopictus* exposed to DDT indicated that most KDT_50_ values from field populations were higher (non overlapping 95% CIs) than those of the reference strain, ATM95, except for NJMer1 and NJBer that showed lower KDT_50_ (Table 4). The two populations from Florida, FL1 and FL2, showed the highest RRs (1.61 and 1.88 respectively) for DDT. For deltamethrin the RRs ranged from 1.13 (NJMer2) to 1.74 (NJMon2) indicating that all the populations were susceptible. Likewise, for phenothrin the KDTs were lower than those of the susceptible strain and for prallethrin the RR_50_ did not exceed 1.18 (FL1). Of note, the two populations from Florida (FL1 and FL2) showed RRs with values of 2.16 and 2.34, respectively, for malathion. The RR_50_ for malathion for the remaining populations were low but significantly higher than 1 and ranged from 1.15 to 1.67.

**Table 4:** Knock down times (min), Resistant ratio, and mortality rates (after 24 h) of *Aedes albopictus* after exposure to insecticides at the diagnostic doses (WHO tube test).

	DDT (4%)			Deltamethrin (0.05%)			Phenothrin (1.5)			Prallethrin (1%)			Malathion (0.8%)		
Population	KDT_50_ (95% CI)	RR_50_	Mortality (%)	KDT_50_ (95% CI)	RR_50_	Mortality (%)	KDT_50_ (95% CI)	RR_50_	Mortality (%)	KDT_50_ (95% CI)	RR_50_	Mortality (%)	KDT_50_ (95% CI)	RR_50_	Mortality (%)
**ATM95**	33 (30–35)	1	100	6 (6.3–6.6)	1	100	8.4 (8.2–8.7)	1	100	1.37 (1.29–1.44)	1	100	23 (22.2–23.8)	1	100
**FL1**	53 (51–56)	**1.61**	72	9.8 (9.6–10.1)	**1.57**	100	7.9 (7.6–8.3)	0.94	100	1.61 (1.46–1.82)	**1.18**	100	49.6 (47.4–52.3)	**2.16**	86
**FL2**	62 (59–68)	**1.88**	54	10.7 (10.4–10.9)	**1.70**	99	7.7 (7.4–8)	**0.91**	100	1.35 (1.28–1.43)	0.99	99	53.5 (50.5–57.6)	**2.34**	80
**NJMon1**	42 (41–44)	**1.28**	100	8.4 (7.9–8.9)	**1.34**	100	6.6 (6.4–6.8)	**0.79**	100	0.81 (0.73–0.88)	**0.59**	99	27.9 (26.9–28.8)	**1.22**	100
**NJMon2**	42 (40–45)	**1.28**	87	11.2 (10.9–11.5)	**1.78**	100	7.6 (7.3–7.9)	**0.9**	100	1.37 (1.25–1.5)	1.04	100	36.3 (35.1–37.5)	**1.59**	95
**NJMer1**	28 (24–30)	**0.83**	95	10.2 (9.4–11)	**1.62**	100	7.1 (6.9–7.4)	**0.85**	100	1.48 (1.29–1.78)	1.08	100	34.3 (33.5–35.2)	**1.5**	96
**NJMer2**	47 (45–49)	**1.4**	100	7.1 (6.8–7.4)	**1.13**	100	6.6 (5.9–7.2)	**0.78**	100	1.12 (1–1.21)	**0.83**	100	26.4 (25.5–27.3)	**1.15**	99
**NJBer**	27 (26–27.3)	**0.8**	99	10.3 (9.9–10.8)	**1.65**	100	8.3 (7.9–8.8)	0.98	100	1.13 (0.99–1.25)	**0.83**	100	38.3 (36–40.4)	**1.67**	93

ATM95: susceptible reference strain. KDT_50_: Knock down time where 50% of the mosquitoes are knocked down (min); RR_50_: Resistant Ratio =  KDT_50_ ATM95/KDT_50_ field population. Significant RRs are shown in bold.

Adult mortality after a 24 h exposure to the pyrethroid insecticides (deltamethrin, prallethrin, and phenothrin) at discriminating doses indicated that, like the ATM95 strain, all the field populations tested can be considered susceptible (99–100% mortality; Table 4). However, the two populations from Florida (FL1 and FL2) showed resistance to DDT (75 and 54% mortality, respectively) and a population from New Jersey (NJMon2) also showed resistance to this organochlorine (87% mortality). In addition, resistance to malathion was found in the two populations from Florida (FL1 and FL2) with 86 and 80% mortality, respectively. Finally, the populations from New Jersey (NJMon2, NJMer1, and NJBer) showed suspected resistance to malathion with 95, 96 and 93% mortality, respectively (Table 4).

### Detoxification enzyme levels

**Figure 2:**
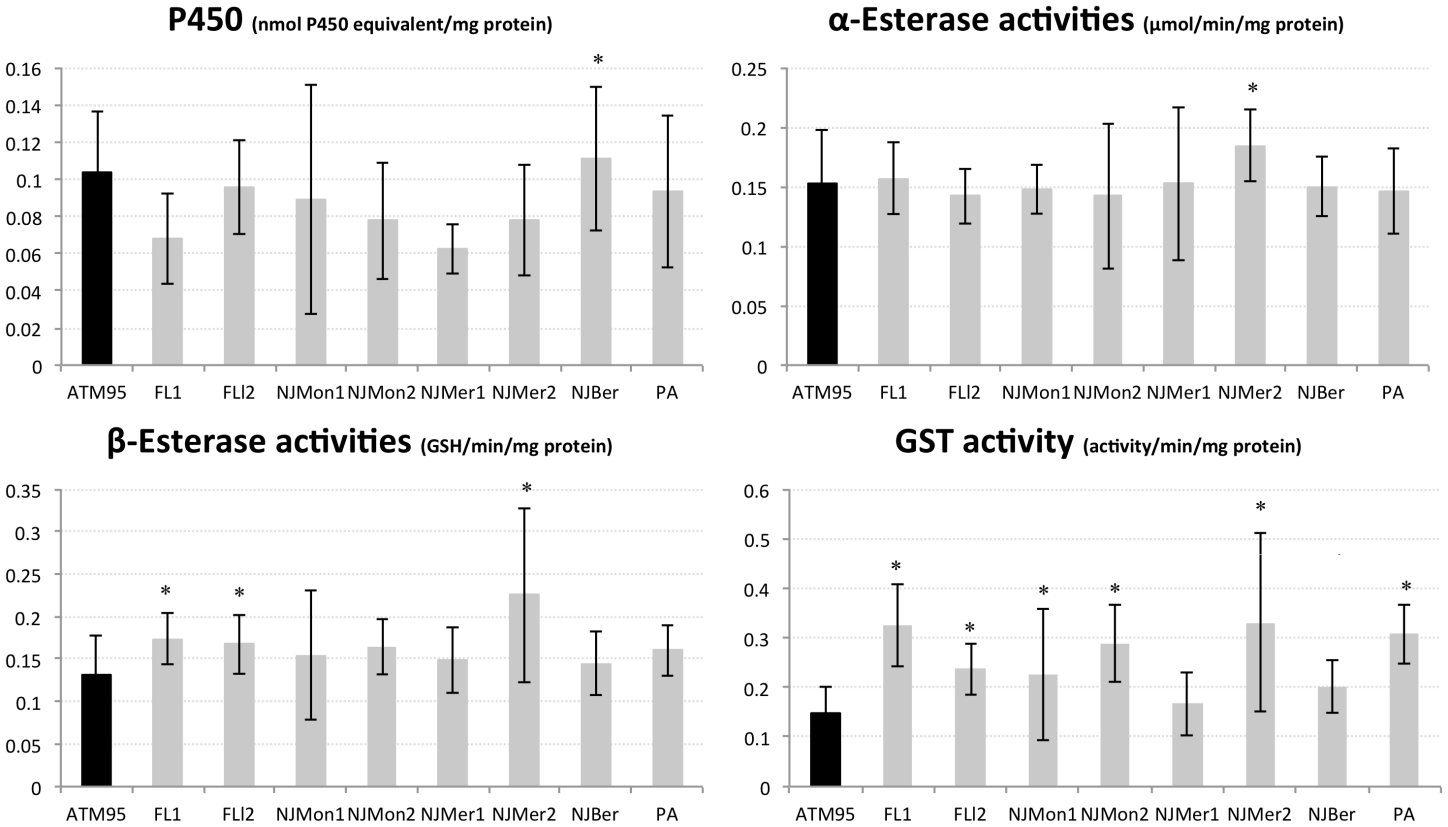
Global amount or activity of detoxification enzymes in adult *Aedes albopictus* from field populations and the laboratory strain (ATM95): cytochrome P450 monooxygenases (P450s), Esterase (α and β-CCEs) and Glutathione-S transferases (GSTs). Sample sizes are 47 specimens/population. Confidence intervals are one standard deviation of the mean. An asterisk (*) denotes significantly up-regulated values compared to the susceptible reference strain ATM95, Tukey-Kramer test.

Comparison of constitutive detoxification enzyme activities between ATM95 and the field strains revealed significant differences in both larval and adult stages (Figure 1 and 2). The P450s levels were significantly higher in larvae from Florida (both FL1 and FL2), NJMon1, and NJMer1 populations. The FL2 and NJMon1 had significantly higher α- and β-ESTs activities and GSTs activities were significantly higher in most populations, particularly in FL1 and FL2, but not in NJMon2 and NJMer1 (Figure 1). In adults, only NJMer2 showed significantly up-regulated P450s, and only NJMer2 had significantly higher α-ESTs activities. The two populations from Florida and NJMer2 had significantly higher β-ESTs activities. Finally, except for NJMer1 and NJBer, all populations had significantly higher GSTs activities (Figure 2) than the susceptible strain.

### Kdr genotyping

We obtained clean sequences of exonic regions in domains II (480 bp), III (exon 1 and 2, 347 bp), and IV (280 bp) of the voltage-gated sodium channel. Of note, in approximately half of the specimens in domains II and III we were not able to span the introns due to the presence of insertions or deletions and therefore we could not obtain both forward and reverse exonic sequences. We compensated by sequencing twice in each direction. Although a few silent mutations at codon positions 2 and 3 were seen, no amino-acid changing mutations were detected in the exons of domains II and IV of the mosquitoes tested. However, in one individual, a mutation was found in domain III at position 1534 (base pair positions are numbered according to the amino acid sequence of the most abundant splice variant of the house fly sodium channel, GenBank accession nos. AAB47605 and AAB47604) where a substitution occurred (TTC to CTC), changing the wild type Phenylalanine into a Leucine. The mutation in residue 1,534 that has been associated with pyrethroid resistance in *Ae. aegypti* is F1,534C, resulting in a Cysteine [62].

### Enzymatic phenotyping of Ache1

All mosquito test populations from New Jersey, Pennsylvania, and Florida showed similar percentages of AChE inhibition with dichlorvos and propoxur compared to the susceptible ATM95 strain (data not shown), indicating they are all of the susceptible type.

## Discussion

The purpose of this study was to evaluate the insecticide resistance status of *Ae. albopictus* populations in several states along the eastern coast of the USA. Insecticides representing the major classes of insecticide (OC, OP, CA, PYR), bio-insecticides (*Bti* and spinosad), and IGRs were used in this study against larvae and adult mosquitoes following WHO protocols. We investigated the possible insecticide resistance mechanisms involved (detoxification enzyme and target site mutations) with biochemical and molecular assays.

For both bioassays and biochemical assays, the eight populations tested were compared to the ATM95 strain, which we first characterized for insecticide susceptibility. The ATM95 strain had similar or higher susceptibilities to the insecticides tested than other *Ae. albopictus* populations used as a reference in previous studies. For example, Ali et al. [26] showed higher LC_50_ for an *Ae. albopictus* strain from Florida maintained for 2 yrs in colony for temephos, *Bti*, methoprene, and pyriproxyfen of 0.01, 0.181, 0.0022, and 0.00011 mg/L respectively, than the ATM95 strain with LC_50_ for the same insecticides of 0.00054, 0.07, 0.00014, and 9.4 10^−6^ mg/L. The susceptible reference strain Ikaken used for the study by Liu et al. [28] presented higher LC_50_ for *Bti*, propoxur, and spinosad (0.1, 3.3, and 0.3 mg/L, respectively) than the LC_50_ of ATM95 (0.07, 1.2, and 0.1 mg/L respectively). Furthermore, the larvae of the ATM95 strain showed higher susceptibility to deltamethrin, permethrin, and malathion than the Ikaken strain or the susceptible strain used by Selvi et al. [45]. In light of these results, we consider the ATM95 as a valid susceptible reference strain for the present study and propose it should be adopted as a reference in future studies of insecticide resistance in temperate *Ae. albopictus*. Reference strains such as the Rockefeller or Bora-Bora used for *Ae. aegypti* studies are essential for the quantification of insecticide resistance across studies [46].

The larval bioassays showed that none of the eight populations examined were strongly resistant to the larvicides tested. Likely because of their specific modes of action, resistance to *Bti*, spinosad, or pyriproxyfen has not been described in mosquitoes, except for a single case of putative resistance to *Bti* in a *Culex pipiens* L. population from New York [47], making these insecticides promising tools for the control of *Ae. albopictus* in the USA. However, we note that spinosad resistance has been reported in several insect pests previously, indicating that it is possible that resistance may occur over time in *Ae. albopictus* if intensive use occurs [48]. Our results showed that temephos was still effective against all the populations tested, although several studies have suggested that temephos resistance selection can develop in *Ae. albopictus* after laboratory selection or prolonged field exposure [49], [50]. Indeed, resistant populations have been detected in South-East Asia, South America, and in Europe, where this larvicide is used against *Aedes* species [16], [19]. The use of temephos for control of *Ae. albopictus* larvae in the USA should therefore be carefully evaluated since adult populations from Florida and New Jersey showed resistance or suspected resistance to malathion (OP). Also, the low but significant resistance to propoxur (CA) exhibited by the Florida and New Jersey populations (RR_50_ >2) should be taken into consideration since cross-resistance is known to occur between OPs and CAs.

Methoprene has been used for vector control in Florida for more than 3 decades [51] and even when *Ae. albopictus* is not been the primary control target in this area, populations may have been exposed to this insecticide and developed tolerance over time. One Florida population showed suspected resistance to both methoprene and pyriproxyfen and the adults showed resistance to the adulticide malathion. Previous authors have reported similar findings in mosquitoes exhibiting high resistance to OPs. Specifically, Marcombe et al. [52] and Andrighetti et al. [53] showed that *Ae. aegypti* populations with high resistance to the organophosphate temephos were less susceptible to pyriproxyfen, indicating a possible cross resistance in mosquitoes between these two insecticides families.

The adult bioassays revealed resistance to malathion in Florida and suspected resistance in New Jersey. Resistance to this insecticide, which is used in space spraying treatments was already a concern for the public health authorities in the 1980's [54] when malathion resistance in *Ae. albopictus* was described in Texas only a few years after *Ae. albopictus* became established. Furthermore, other studies report resistance to malathion in populations from Louisiana, Illinois, Alabama, and additional locations in Texas [25], [27], [28]. Worldwide *Ae. albopictus* resistance to malathion has been extensively reported in Asia, the presumed origin of the USA populations of this species, since the 1960's [55], and it is possible that the introduced populations were already resistant. However, since malathion and other OPs are still being used for mosquito control in the USA, it is also possible that resistance developed locally and is being maintained in this region.

All the populations were susceptible to the three pyrethroids tested at the diagnostic doses. Prallethrin and phenothrin are the components of the Duet formulation that showed promising efficacy in ultra-low volume adulticide applications against *Ae. albopictus* [14]. All the populations were also susceptible to deltamethrin, showing that this insecticide can still be an effective tool for *Ae. albopictus* control. However deltamethrin or pyrethroid resistance has already been detected in China, Japan, and South-East Asia [16], [19], [22], [56] and also more recently in Florida and Alabama, USA [28].

Although we were initially surprised to detect DDT resistance in Florida populations of *Ae. albopictus*, DDT resistance is widespread in *Ae. albopictus* populations worldwide especially in Asia. Since the 1960's very high levels of resistance have been reported from India to the Philippines and from China to Malaysia [18], [22]. So as for malathion resistance, it is also likely that the selection for resistance may have occurred in Asia, prior to USA introductions. However, since the use of DDT was terminated in the USA in 1972, before the introduction and establishment of *Ae. albopictus*, the observed levels of resistance in Florida may be explained by a regular exposure of the populations to pyrethroids or other xenobiotics that have the same mode of action as DDT. Alternatively, it is possible that DDT resistance in these populations does not impact fitness and therefore is simply being maintained neutrally or finally, that there have been more recent introductions of DDT resistant *Ae. albopictus* from Asia (Fonseca et al. unpublished data). This last scenario is supported by the study of Kamgang and colleagues [23] that reported DDT resistance in recently introduced populations in Cameroon. The high levels of resistance against DDT found in Florida and the suspected resistance in the populations from New Jersey also underscore the threat of pyrethroid resistance in USA *Ae. albopictus*. Cross resistance mechanisms between DDT and pyrethroids can negatively impact control strategies.

Regarding the various mechanisms of insecticide resistance, we found significant differences in detoxification enzyme activities in several USA resistant *Ae. albopictus* populations suggesting the involvement of metabolic based resistance mechanism. The malathion resistant populations from Florida and New Jersey showed significantly over-expressed *β*-ESTs and GSTs, which include two detoxification enzyme families known to play a role in organophosphate resistance in mosquitoes [32]. However, because several studies have showed that carboxylesterases do not play a role in resistance to organophosphate in *Ae. albopictus* [45], [57], it remains unclear whether one or both of the enzyme families are involved in the resistance at the adult stage. Complementary studies with the use of specific enzyme inhibitors should be implemented to discriminate their roles in malathion resistance in the USA *Ae. albopictus*.

Larvae from Florida populations showed the highest RR_50_ against propoxur but were not resistant to temephos, confirming the absence of insensitive AChE responsible for the cross-resistance between OP and carbamates in mosquitoes. Of note, insensitive AChE was recently detected in *Ae. albopictus* populations in Malaysia [20], underscoring the importance of regular monitoring of this mechanism in the USA. All the populations tested showed a reduced susceptibility against propoxur and all had a significantly increased amount of P450s. It is therefore possible that P450s may be involved in carbamate resistance in *Ae. albopictus* as in other mosquito species [58].

One population from Florida showed significant resistance against the two IGRs, methoprene and pyriproxyfen. The same population also presented over-expressed P450s, ESTs, and GSTs. The P450s are primarily involved in pyrethroid (DDT) resistance and may also be involved in IGR resistance in insects [59]. Indeed, recently the product of the *Ae. aegypti* CYP6Z8 detoxification gene, belonging to the P450s family, was shown to metabolize pyriproxyfen [60]. There are many reports demonstrating elevated P450 activity in insecticide resistant mosquitoes, frequently in conjunction with altered activities of other enzymes [32]. The global overexpression of the four detoxification enzyme families in *Ae. albopictus* from Florida may therefore be leading to a reduced susceptibility to IGRs.

In all populations that presented DDT resistance, GSTs were significantly overexpressed in the adults. This is not surprising since GST-overexpression is the major metabolic mechanism inducing DDT resistance [32], [61] and the involvement of the DDT-dehydrochlorinase, now classified in the GST family, has been demonstrated in DDT resistant *Ae. albopictus* populations in China. The GSTs probably play an important role in DDT resistance in *Ae. albopictus* in the USA and this should be confirmed by the use of synergists in future studies. The other possible mechanism involved in DDT but also in pyrethroid resistance is a target site modification such as the *kdr* mutation [29]. Although none of the populations showed resistance to pyrethroids we identified a previously unknown amino acid polymorphism (F1534L) in domain III of the VGSC, in a location known to be associated with pyrethroid resistance in *Ae. aegypti* [62], in one of the Florida specimens. Kasai et al. [43] found at the same location a mutation leading to a cytosine in *Ae. albopictus* collected from Singapore (F1534C) but besides the fact that the area where the colony originated was treated with permethrin in the 1980s, there was no information about the current resistance status of this population against pyrethroids. This is the first time such a mutation is detected in *Ae. albopictus* and given the increasing use of pyrethroids for vector control in the USA [63], [64] it is important to pursue studies on the global distribution of this allele and its involvement in pyrethroid resistance.

In conclusion, our studies have generated a fully characterized susceptible reference population for temperate *Ae. albopictus*, ATM95, which is available upon request from dinafons@rutgers.edu. We have also uncovered a complex landscape of populations of *Ae. albopictus* in the USA that are broadly susceptible to larvicides and adulticides. Unexpectedly, we found significant resistance to DDT in two Florida populations and in a New Jersey population. We also found resistance to malathion, an organophosphate, in Florida and suspected resistance in New Jersey plus suspected resistance to several insect growth regulators. Several detoxification enzyme families seemed to be involved in resistance as well, but further studies with the use of synergists should be performed to confirm these findings. All populations tested were fully susceptible to pyrethroids, however, we identified a previously unknown amino acid polymorphism (Phe → Leu) in domain III of the VGSC, in a location known to be associated with pyrethroid resistance in *Ae. aegypti*. We developed a rapid diagnostic PCR to detect this mutation (Marcombe and Fonseca unpublished data) but further studies should be conducted to confirm its implication in DDT/pyrethroid resistance and to assess the frequency of this mutation in *Ae. albopictus*.

This study showed standard larvicides and pyrethroids used for mosquito control are still effective against USA populations of *Ae. albopictus*, but it also demonstrates the importance of research on insecticide resistance and the constant need to develop new tools, new insecticides, and innovative strategies to prevent the development of insecticide resistance in these critical vectors of human diseases. Other strategies such as control using genetically modified male mosquitoes [65], or the use of *Wolbachia* to block disease transmission [66] are very promising because they do not use insecticides but the cost-effectiveness of these strategies and their long term success should be evaluated when compared with conventional control methods.
